# Infectious Disease Outbreak Associated With Supplementary Feeding of Semi-domesticated Reindeer

**DOI:** 10.3389/fvets.2019.00126

**Published:** 2019-04-18

**Authors:** Morten Tryland, Ingebjørg H. Nymo, Javier Sánchez Romano, Torill Mørk, Jörn Klein, Ulrika Rockström

**Affiliations:** ^1^Arctic Infection Biology, Department of Arctic and Marine Biology, UiT The Arctic University of Norway, Tromsø, Norway; ^2^Section for Pathology, Norwegian Veterinary Institute, Tromsø, Norway; ^3^Department of Nursing and Health Sciences, Faculty of Health Science, University of South-Eastern Norway, Kongsberg, Norway; ^4^Kungsängens Gård, Farm and Animal Health, Uppsala, Sweden

**Keywords:** alphaherpesvirus, contagious ecthyma, *Fusobacterium*, parapoxvirus, supplementary feeding, zoonosis

## Abstract

Supplementary winter feeding of semi-domesticated reindeer (*Rangifer tarandus tarandus*) has become more common in Sweden and Norway due to reindeer pasture fragmentation and climatic conditions. With increased corralling and feeding, often associated with animal stress, increased animal-to-animal contact, and poor hygienic conditions, an altered range of health challenges and diseases may emerge. An outbreak of three different infectious diseases appeared simultaneously in a reindeer herd in Norrbotten County, Sweden. The animals were corralled and fed silage. Several animals in poor body condition stopped eating, with drool and discoloration of the hair coat around the mouth. There were large, black, necrotic lesions on the tongue and gingiva, with holes perforating the chin, indicative of oral necrobacillosis and *Fusobacterium* spp. infection. Simultaneously, animals were seen with proliferative lesions in the oral mucosa and on the lips, characteristic of contagious ecthyma and Orf virus infection. Furthermore, three animals had keratoconjunctivitis suggesting exposure to cervid herpesvirus 2 (CvHV2) and possibly secondary bacterial infections. DNA specific for *Fusobacterium necrophorum* and ORFV was detected in relevant tissue samples. Antibodies against CvHV2 were detected in 10 of 13 diseased and in four of 11 apparently healthy reindeer. Nine animals were found dead or were euthanized during the outbreak. Health risk factors associated with feeding and corralling may severely impact animal welfare and the herder's economy, and may represent an underestimated cost when replacing natural grazing with feeding.

## Introduction

In Sweden and Norway, semi-domesticated reindeer (Eurasian tundra reindeer; *Rangifer tarandus tarandus*) are for most part of the year free-ranging, grazing on vast, and remote mountain pastures. Feeding was traditionally conducted only when animals were held for draft and milking, and if the herd had to be corralled over a number of days. During the last decades, however, feeding has become more common (1). Reindeer are fed after rain-on-snow events, that create ice-locked pastures, and for gathering and protection against predators. However, reindeer are also increasingly being fed on a more regular basis, as a supplement to natural pastures due to restrictions on pasture resources. Feeding during late winter and early spring, is especially relevant for pregnant females at a point when energy reserves are diminishing (2, 3). Since knowledge on how to successfully feed reindeer has increased, along with the availability of suitable reindeer feed (1, 4), a trend of giving economic compensation for feeding is increasingly offered to herders when the reindeer pastures need to be exploited for other purposes, such as establishing new infrastructure. Corralling and feeding reindeer full daily rations over a period of time is often associated with stress, increased animal-to-animal contact and challenging hygienic conditions (1), factors that together, compounded by poor feed quality, may facilitate the appearance of diseases.

Orf virus (ORFV; genus *Parapoxvirus*, family *Poxviridae*) is distributed worldwide in sheep and goats, but may infect a wide range of wild ruminant species (5) and is also zoonotic, causing painful skin lesions in man (6). In reindeer, ORFV cause the disease contagious ecthyma (syn. contagious pustular dermatitis), with characteristic proliferative “cauliflower-like” lesions at the muco-cutaneous junctions of the mouth and nose, as well as in the oral mucosa (7). Contagious ecthyma was reported in reindeer under natural herding conditions in Sweden in 1973 (8) and in Norway in 2001 (9), but has rarely been diagnosed since in these countries. This sporadic occurrence is in contrast to the situation in Finland, where it has been diagnosed virtually every year since the winter of 1992–1993 (7), when about 400 reindeer succumbed and about 2,800 were affected (10). Later outbreaks in Finland have been associated with pseudocowpoxvirus (PCPV), a parapoxvirus with a cattle reservoir (11).

Necrobacillosis is caused by *Fusobacterium necrophorum*, a Gram-negative rod-shaped, toxin-producing bacterium (12). Digital necrobacillosis was a well-known and feared disease in semi-domesticated reindeer in the seventeenth and eighteenth century (13, 14). Necrobacillosis is characterized by severe lesions on the hooves (digital form) and in the gastrointestinal tract (oral form), sometimes causing severe disease outbreaks affecting many animals (15, 16). The bacterium is a part of the normal rumen and fecal microbiota in reindeer (17), and disease outbreaks were often seen in animals corralled for milking, and under wet and poor hygienic conditions (18, 19). Digital necrobacillosis is rarely reported from Fennoscandian reindeer herds today, but the oral form seems to have become more common (16, 20). *Fusobacterium necrophorum* is also increasingly reported as a zoonosis (21).

The reindeer alphaherpesvirus, cervid herpesvirus 2 (CvHV2), is enzootic in the Fennoscandian reindeer population (22–24). The virus may cause upper respiratory tract infection in reindeer, and is also transferred from mother to fetus, with a potential for abortion and weak borne calves (7, 22). Mucosal lesions in the mouth and eyes caused by CvHV2 may facilitate secondary infections by opportunistic pathogens, such as ORFV, *Fusobacterium necrophorum*, and other species of bacteria (7, 25–27).

## Case Report

We present a disease outbreak in Norrbotten County, Northern Sweden, in March 2016, in a herd of semi-domesticated reindeer corralled for winter feeding, displaying necrotizing, and proliferative mucosal and skin lesions as well as eye infections.

The reindeer herd was corralled and fed silage due to ice-locked winter pastures and acute food shortage. After 1 month in the initial corral the herder registered that one calf (<1 year old) in good body condition started to cough, with increasing severity. The calf stopped eating and died a few days later (March 20th) (Table 1, 1H). The herd was thereafter herded to the calving ground. Upon arrival in the second corral (March 29th), a 2-year-old reindeer stopped eating and had a severely foul odor from the mouth with a perforation through the chin. Upon closer inspection, large wounds were found in the oral mucosa of the palate and the gingiva and the animal was euthanized (Table 1, 2H). The herder subsequently registered more animals that were drooling with eating difficulties. On April 3rd, the owner found a calf on the mountain pasture, in good condition, but with a severe necrotizing lesion on the tongue, with large parts of the tongue missing. This animal was euthanized and the tongue secured for diagnostic analyses (Table 1, 3H). On the same day, an adult female that had lost weight and recently aborted her fetus, was found with a hole in the chin and was euthanized (Table 1, 4H).

**Table 1:** Overview of clinical diagnoses and diagnostic findings in 32 semi-domesticated Eurasian tundra reindeer (*Rangifer t. tarandus*) during a disease outbreak among corralled and supplementary fed animals in Norrbotten County, Sweden, spring 2016.

**ID**	**Date dd.mm 2016**	**Location**	**Sex**	**Age**	**Parapoxvirus**		**Necrobacillosis**		**Eye lesions**	CvHV2^*^ serology	**Abortion**	**Found dead** **(D)** **euthanized** **(E)**
					**Lesions**	**PCR**	**Lesions**	**PCR**				
1H	20.03	Initial corral	–	C	NI		1					D
2H	29.03	Second corral	M	1	NI		1					E
3H	03.04	MP	M	C	NI	1 (tissue)	1	1 (tissue)				E
4H	03.04	MP	F	>5	NI		1				1	E
5H	06.04	DC	M	C	NI		1					E
6H	08.04	MP	F	>5	NI		1					E
7H	09.04	DC	F	C	NI		1					D
8H	09.04	DC	F	>5	1	0 (tissue)	1	1 (tissue)			1	E
1V	05.04	HC	M	C	0		0		1	1		
2V	05.04	HC	M	C	0		0		0	0		
3V	05.04	HC	M	C	0		0		0	0		
4V	05.04	HC	F	>5	0		0		0	0		
5V	05.04	HC	F	>3	0		0		0	0		
6V	05.04	HC	M	C	0		0		1	0		
7V	05.04	HC	M	C	0		0		0	0		
8V	05.04	HC	M	2	0		0		0	1		
9V	05.04	HC	M	3	0		0		0	1		
10V	05.04	HC	F	C	0		0		0	0		
11V	05.04	HC	F	3	0		0		1	1		
12V	05.04	DC	M	2	1	0 (swab)	0	0 (swab)	0	1		
13V	05.04	DC	M	2	0		0		0	1		
14V	05.04	DC	F	C	1	0 (swab)	0	0 (swab)	0	1		
15V	05.04	DC	F	C	1	0 (swab)	0	0 (swab)	0	0		
16V	05.04	DC	M	C	1	0 (swab)	1	1 (swab)	0	0		
17V	05.04	DC	M	2	1	0 (tissue)	1	1 (tissue)	0	1		E
18V	05.04	DC	M	>1	1	0 (swab)	0	0 (swab)	0	1		
19V	05.04	DC	M	C	1	0 (swab)	0	1 (swab)	0	0		
20V	05.04	DC	F	2	0		0		0	1		
21V	05.04	DC	F	>5	0		0		0	1		
22V	05.04	DC	M	2	1		0		0	1		
23V	05.04	DC	F	>5	0		0		0	1		
24V	05.04	DC	F	2	1	0 (swab)	0	0 (swab)	0	1		

*ID: H, evaluated by herder; V, evaluated by veterinarian. Location: Initial corral, Second corral (after translocation), MP: Mountain pasture, DC: Diseased corral, HC: Healthy corral*.

*Clinical evaluation and laboratory results: NI, Not investigated; 1, positive (shaded grey); 0, negative. Sex: F = female, M = male. Age: C = calf of the year*.

* *Swab samples from eye (n = 24), nose (n = 24), and vagina (n = 7) were all negative with regards to CvHV2-specific DNA (PCR; data not shown)*.

Due to the severity of the clinical signs observed, the reindeer herder consulted veterinary services. When the veterinarian arrived (April 5th), the herder had divided the animals in one “healthy corral” (HC) with animals with no apparent disease signs and one “diseased corral” (DC) with animals with various disease signs. The rest of the herd was looked after on the surrounding mountain pasture (MP). Clinical cases that were investigated, sampled, and analyzed are presented in Table 1. After the veterinary visit, the herder registered four more animals with oral clinical signs (Table 1, 5H−8H) during the period April 5th−9th. Except for the first case (March 20th), the main disease outbreak was registered and evaluated in the new corrals during a 12 days period (March 29th–April 9th).

**Figure 1:**
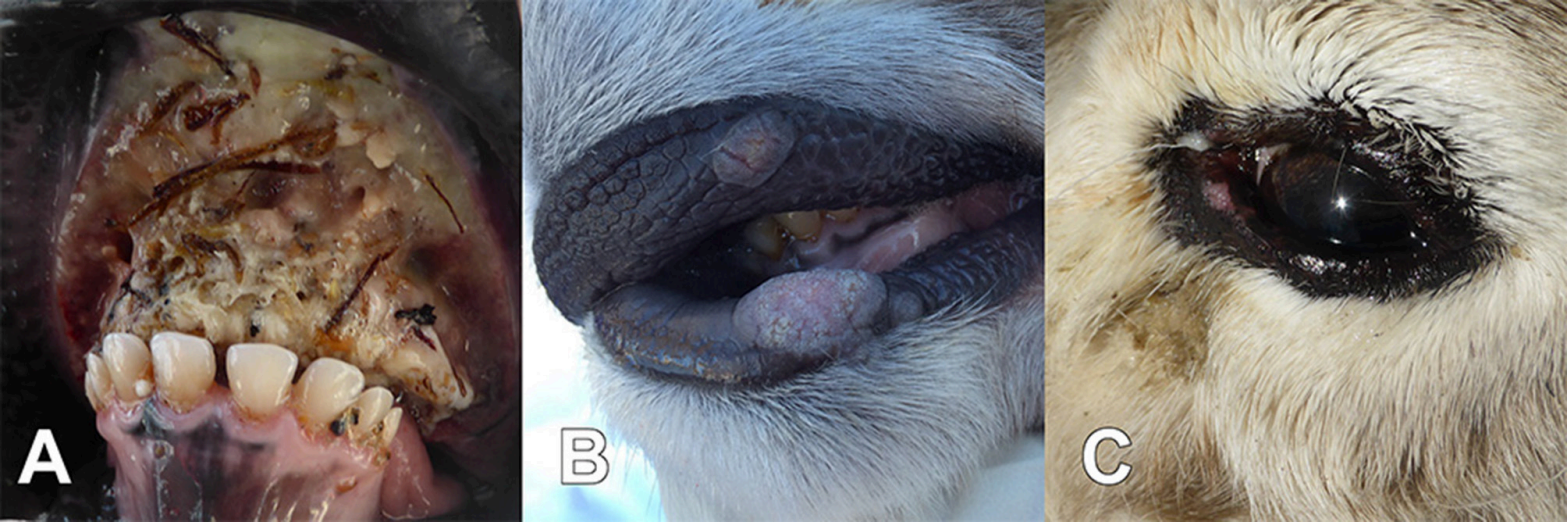
During a disease outbreak among semi-domesticated Eurasian tundra reindeer (*Rangifer tarandus tarandus*), animals found dead or euthanized for animal welfare reasons had necrotizing lesions in the tongue, gingiva, and the oral mucosa, characteristic for necrobacillosis **(A)** (Table 1, 17V), and clinical signs typical of contagious ecthyma, with proliferative lesions on the lips and in the oral mucosa **(B)** (Table 1, 18V). In addition, eye infections were observed in three of the animals **(C)** (Table 1, 11V).

Samples obtained from the animals evaluated by the herder (*n* = 8; March 20th–April 9th) were the tongue from animal 3H and the head from animal 8H. In addition, the veterinarian carried out a thorough clinical investigation of 11 reindeer in HC and 13 in DC with multiple samples taken (blood, swab samples from nose, eye and vagina, and from lip/mouth lesions). Two of the 32 affected and examined reindeer were found dead and seven were euthanized. All had lesions typical of oral necrobacillosis, i.e., excessive salvation with a foul smell, problems in chewing and swallowing food, and necrotic lesions in the mouth (Figure 1A). In addition, the veterinarian found clinical signs of contagious ecthyma in nine animals, i.e., papules, pustules, and cauliflower-like proliferations around the mouth (Figure 1B). Three animals (Table 1, 8H, 16V, and 17V) had clinical signs of both diseases (Table 1). Bilateral eye infections (i.e. keratoconjunctivitis, pus discharge, and peri-orbital edema) were observed by the veterinarian in three animals (Table 1, 1V, 6V, and 11V) (Figure 1C). These three had all been corralled in HC and had no clinical signs of necrobacillosis or contagious ecthyma.

A serological investigation (28, 29) revealed antibodies against alphaherpesvirus in 10 of 13 animals from DC and in four of 11 animals from HC (Table 1). Six of the nine animals with contagious ecthyma and two of the four animals with eye infections had antibodies against alphaherpesvirus.

Swabs from lesions (skin and oral mucous membrane) were inoculated on sheep blood agar, incubated under anaerobic conditions at 37°C, and checked for bacterial growth after 24 and 48 h. *Fusobacterium necrophorum* was not cultivated from any of the bacteriology swab samples from necrotized tissues.

**Figure 2:**
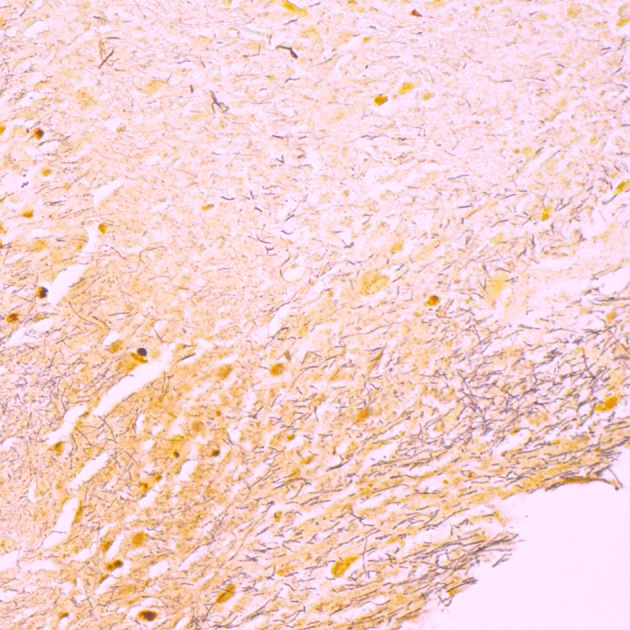
Micrograph showing Warthin-Starry staining of a section of paraffin embedded tissue of a lesion in the hard palate of the submitted head (Table 1, 3H), demonstrating necrotic masses with cell debris and large amounts of long, filamentous bacterial rods (stained brown).

Tissue samples from the submitted head (Table 1, 8H) and tongue (Table 1, 3H) were subjected to macroscopic and microscopic investigations. The head (Table 1, 8H) had a mucosal lesion covering about one third of the rostral part of the hard palate, consisting of yellow and grayish, soft necrotic tissue with hyperemia. The lesion was clearly demarcated from normal tissue and did not include the lips. A tissue sample from this lesion was fixed in 10% formalin for histopathology and stained with the Hematoxylin-Eosin (HE) and Warthin-Starry silver nitrate techniques. Microscopic examination showed large necrotic masses and cell debris, loss of epithelium of the mucosa and hyperemia, with neutrophil granulocytes, and fibrin in the submucosa below the necrotic masses. The Warthin-Starry staining revealed large amounts of long bacterial rods in the necrotic masses (Figure 2). The submitted tongue (Table 1, 3H) displayed a deep wound on the dorsal side and about half of the rostral part of the tongue had been lost. The affected tissue had mild hyperemia and was soft with a thin, pale demarcation line toward normal tissue. The lesion itself seemed less acute and was likely in the repair phase.

DNA was extracted from muscular tissue from the mouth of three reindeer with necrotizing lesions (Maxwell 16® Tissue DNA purification kit; Promega, Madison, WI, USA) and from swab samples of the mucosal membrane of the eye (*n* = 24), nose (*n* = 24), vagina (*n* = 7), and mouth/lip lesions (*n* = 7) (Table 1) (Maxwell 16® Buccal Swab LEV DNA purification kit; Promega) as described previously (30). PCRs targeting parapoxvirus, *B2L* and *GIF* gene regions, were conducted (31) on seven swab samples from mucosal membranes and three tissue samples from animals with mouth mucosal lesions consistent with contagious ecthyma (*n* = 9) and necrobacillosis (*n* = 4). Extracted DNA from a skin lesion of a goat with contagious ecthyma was used as a positive control. Parapoxvirus-specific DNA was detected in tissues of the tongue with necrobacillosis lesions but without clinical signs of contagious ecthyma (Table 1, 3H).

To target DNA specific to *F. necrophorum*, a real-time quantitative PCR (qPCR) was performed (32) on the same samples as investigated with the parapoxvirus PCRs (Table 1). DNA extracted from *F. necrophorum* isolated from a wild reindeer (*R. t. tarandus*) with digital necrobacillosis (Ref. no.: 2016-4-11473, Norwegian Veterinary Institute, Oslo) was used as a positive control. DNA specific for *F. necrophorum* was detected by qPCR in all necrotic tissues sampled from the mouth of animals 3H, 8H, and 17V with threshold cycle (C_T_) values varying from 15.6 to 27.2. *Fusobacterium necrophorum* DNA was also detected from swab samples from lesions on the lip of animal 16V and in the mouth of animal 19V (C_T_ values of 25.7 and 29.2, respectively).

To target CvHV2-specific DNA, a qPCR was performed (33, 34). DNA from a CvHV2 isolate obtained from a reindeer with IKC (26) was used as positive control. CvHV2-specific DNA was not detected in any of the swab samples from eye (*n* = 24), nose (*n* = 24), or vagina (*n* = 7).

**Figure 3:**
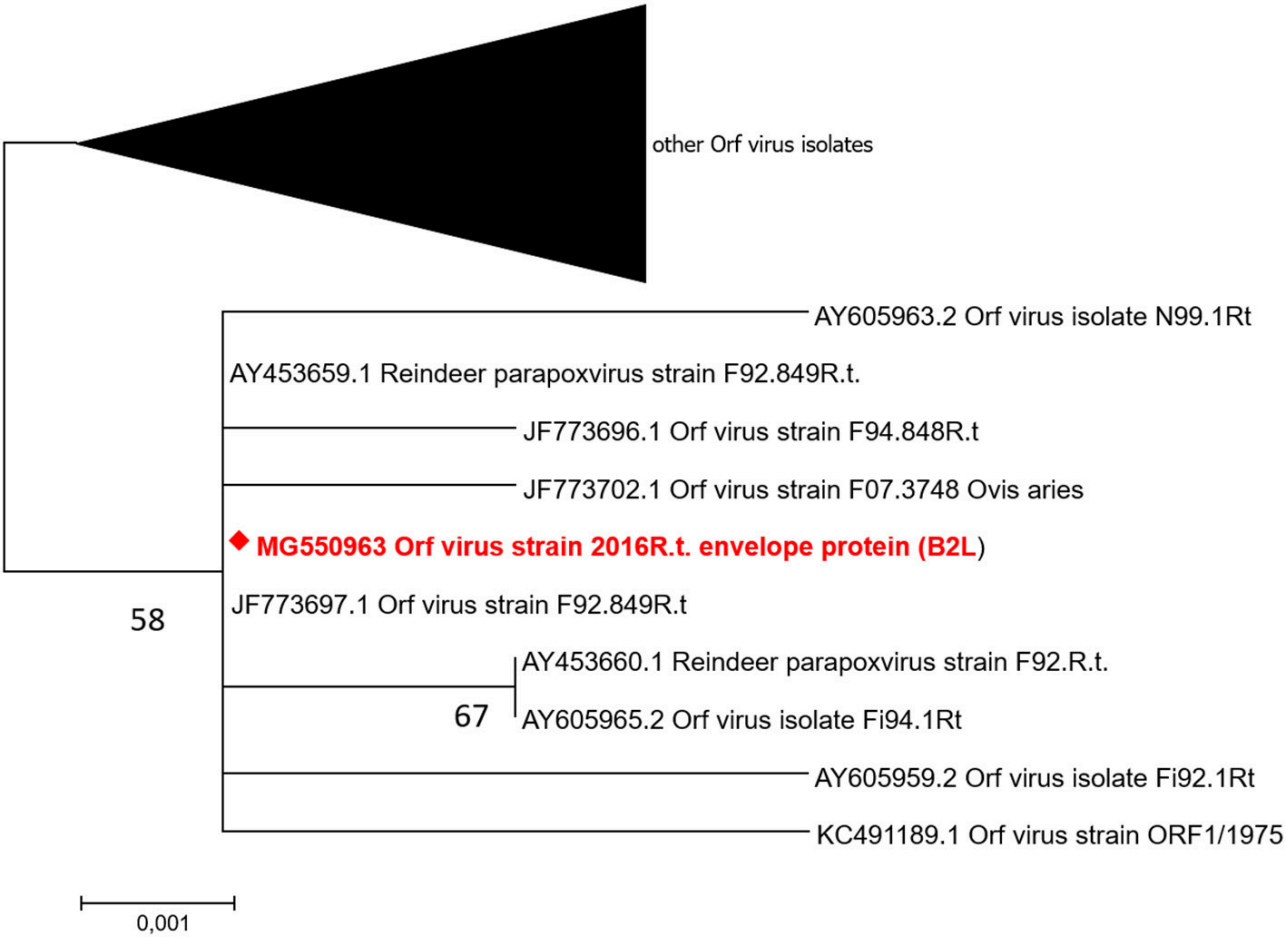
Molecular phylogenetic analysis of ORFV major envelope protein gene (*B2L*). The isolate from this study (diamond; MG550963) was clustering with six ORFV reindeer isolates from Finland (from 1992 to 1994) and one ORFV reindeer isolate from Norway (all labeled R. t.), as well as two ORFV isolates from sheep. The tree is drawn to scale with branch lengths measured in the number of substitutions per site.

The parapoxvirus and *F. necrophorum* PCR products were sequenced (BigDye® Terminator v3.1 cycle sequencing kit; Applied Biosystems, Norway) in an Applied Biosystems 3130 XL Genetic Analyzer (Applied Biosystems) after removal of unused dNTP and primers (ExoSAP-IT™; Amersham Pharmacia Biotech, Sweden). To compare parapoxvirus sequences (GenBank Accession numbers *B2L*: MG550963, *GIF*: MG582651) with similar gene sequences (GenBank), phylogenetic analysis was conducted (Maximum Likelihood; T92) (35). The tree with the highest log likelihood (*B2L*: −1113,02, *GIF*: −1217,86) was chosen. The analysis involved 99 nucleotide sequences and 522 nucleotides in the final dataset for *B2L* gene, and 78 nucleotide sequences, and a total of 292 nucleotides for the *GIF* gene. The codon positions included were 1st+2nd+3rd+Non-coding for both genes, and all positions containing gaps and missing data were eliminated. Evolutionary analyses were conducted in MEGA7 (36). The phylogenetic trees are displayed in Figure 3 (*B2L*) and Supplementary Figure 1 (*GIF*), respectively. For the *B2L* gene, the phylogenetic topology shows that the isolate from this outbreak (MG550963) clustered together with Finnish and Norwegian ORFV isolates from reindeer. The *GIF* gene sequences obtained from this outbreak clustered with one reindeer isolate from Norway (obtained 1999) (9) as well as isolates from sheep (Supplementary Figure 1).

Amplicons generated by the *F. necrophorum* PCR (GenBank accession numbers MH549407, MH549408, MH549409, MH549410, MH549411) and a BLAST analysis revealed a 97–99% nucleotide homology with the *F. necrophorum* subsp. *necrophorum* strain NCTC 10576 RNA polymerase ß-subunit gene (GenBank: AY519655), confirming the PCR results. Phylogenetic studies revealed little relevant information (data not shown).

## Discussion

The diagnosis of contagious ecthyma, necrobacillosis, and transmissible eye infections are often based on clinical observations only, and this is the first report of all three diseases being verified and evaluated during the same disease outbreak, indicating that these infections may have interacted with each other.

In contrast to *Fusobacterium* spp., which is present in the intestinal microbiota of reindeer, and to CvHV2 which is enzootic in the Fennoscandian reindeer herds, it is assumed that parapoxvirus, such as ORFV, is not enzootic in Norwegian and Swedish reindeer populations. Parapoxvirus-specific DNA has been detected (PCR) in reindeer carcasses with no clinical signs of contagious ecthyma and from a region of Norway (Finnmark County) from which the disease has been reported in sheep but never in reindeer (37). However, due to the sporadic appearance of the disease in Norway and Sweden, it seems likely that ORFV in the outbreak described here may have been introduced to the reindeer herd, via direct contact with sheep or goats, or through a contaminated environment (e.g. fences and animal transport vehicles previously being used for small ruminants). The incubation period after inoculation of ORFV into slightly scarified oral mucosa of reindeer has been shown to be only 2–7 days (38), but the timeline of contagious ecthyma during this outbreak indicates that the virus was introduced and transmitted to other animals over a longer period of time. The phylogenetic analysis of the *B2L* gene (Figure 3) of ORFV from this outbreak showed a high degree of homology to ORFV isolates originating from Finnish reindeer. In contrast, the phylogeny based on the *GIF* gene (Supplementary Figure 1) confirmed the Fennoscandian origin of the virus isolate from this outbreak, although it clustered with one Norwegian reindeer isolate and sheep isolates, whereas other reindeer isolates (Norway and Finland) were grouped in two different clusters, separated from each other. These results support the assumption that there is no specific reindeer ORFV.

Since *F. necrop*horum is excreted in the feces, corralling of animals over a longer period of time, and under poor hygienic conditions, will increase their exposure to this bacterium. Oral necrobacillosis was the first disease to be recognized by the herder. It is, however, likely that ORFV has been present over some time, producing lesions that facilitated the establishment of the *F. necrophorum* infection. In fact, four of the cases of oral necrobacillosis were recognized after the veterinary investigation and sampling, when all nine cases of contagious ecthyma were diagnosed. *Fusobacterium necrophorum* is usually a secondary invader which needs a skin wound or mucosal abrasion to be able to establish an infection (39). Mucosal lesions can be caused mechanically, from harsh fodder such as hay from late harvests of grass, or by animals eating ice-crusted snow. In young animals, the gingiva can be traumatized due to the eruption of new teeth, since the permanent set of teeth is not present until the age of 28–30 months (19). Five of the 10 animals clinically affected by oral necrobacillosis were calves (<1 year), one yearling, one 2-year-old, and three >5 years of age. Erupting teeth may thus have been relevant for the young animals, but not for the three older animals.

Lesions in the mucosa can also be caused by viruses, such as CvHV2 (25) and parapoxvirus (ORFV) (9). Our findings seem to indicate that ORFV infections were in fact the first to appear, damaging the oral mucosa and contributing to a secondary infection with *F. necrophorum*, as previously suggested (40). Alternatively, alphaherpesvirus may have been the first pathogen, paving the way for other pathogens (25–27), such as ORFV and *F. necrophorum*. The reindeer alphaherpesvirus establishes life-long and latent infections. Reactivation form latency, in response to certain stimuli such as stress, has been shown experimentally, by provoking immunosuppression using glucocorticosteroids (24, 41). Fourteen of the 24 animals (58%) from which blood samples were obtained had alphaherpesvirus antibodies, a prevalence comparable to previous serological screenings of reindeer (22). Interestingly, five of the 11 animals with contagious ecthyma, and from which a blood sample was available, had antibodies against alphaherpesvirus, but none of the eye, nose or vaginal swabs were PCR positive for CvHV2. Furthermore, none of the three animals with eye infections had oral necrobacillosis or contagious ecthyma. The presence of antibodies but absence of viral shedding on the mucosal membranes could indicate that alphaherpesvirus may have participated in the initial stages of the outbreak, and may have entered the latency phase, with no virus shedding at the time the diseased animals were sampled.

Pasture fragmentation due to increasing establishment of infrastructure in areas that traditionally have been used as reindeer pastures is a serious threat to reindeer herding in Fennoscandia (42, 43). Predicted climate change scenarios for arctic and sub-arctic regions indicate higher winter temperatures and increased frequency of rain-on-snow events and freeze-thaw cycles (44, 45). Restricted availability of reindeer pastures, combined with economic support for feeding as a compensation for lost pasture land, will further contribute to increasing feeding practices. This will challenge the traditional reindeer herding, and may also contribute to altered health and disease challenges. Disease outbreaks represent not only animal welfare challenges, but also extra costs and labor for the herders, both factors that may be under-estimated when replacing natural grazing with feeding.

## Ethics Statement

No specific ethical permissions were required for this study. Sampling of the animals was conducted during clinical evaluation and for medical reasons with the objective of determining the cause of the clinical outbreak prior to treatment, with the approval of and in collaboration with the reindeer herder.

## Author Contributions

UR was contacted by the herder and organized the sampling. IN conducted the sampling and some of the analyses. MT supervised sampling and analyses. TM carried out the bacteriology cultivation, pathology, and histopathology. JSR conducted real time PCR. JK conducted phylogenetic analysis. MT and IN wrote the first draft of the manuscript. All authors contributed to the manuscript and accepted the submitted version.

### Conflict of Interest Statement

The authors declare that the research was conducted in the absence of any commercial or financial relationships that could be construed as a potential conflict of interest.

## Supplementary Material

Supplementary Figure 1 Molecular phylogenetic analysis of the ORFV GM-CSF/IL-2 inhibition factor gene (*GIF*). The reindeer virus isolate from this study (diamond) was clustering with an ORFV isolate from reindeer in Norway (N99 1Rt), but also ORFV isolates from sheep, whereas the Finnish and the remaining Norwegian reindeer isolates constituted two other and separate clades. The tree is drawn to scale, with branch lengths measured in the number of substitutions per site.
