# Supplementary figures and images for: Infectious Disease Outbreak Associated With Supplementary Feeding of Semi-domesticated Reindeer
Source: Front Vet Sci. 2019 Apr 18;6:126. doi: 10.3389/fvets.2019.00126 (PMC6482261; doi:10.3389/fvets.2019.00126)

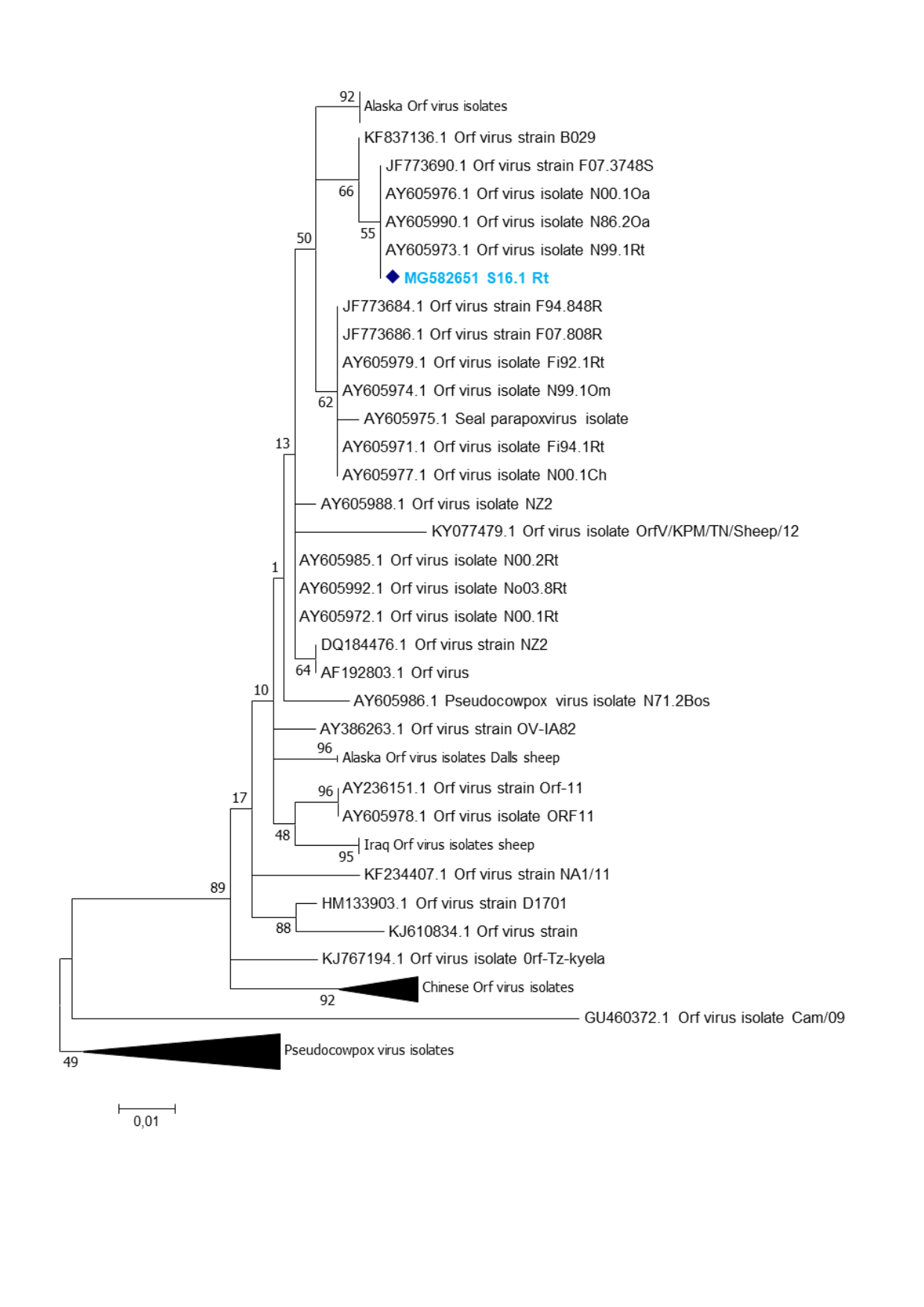

Supplement: Supplementary Figure 1 — Molecular phylogenetic analysis of the ORFV GM-CSF/IL-2 inhibition factor gene (GIF). The reindeer virus isolate from this study (diamond) was clustering with an ORFV isolate from reindeer in Norway (N99 1Rt), but also ORFV isolates from sheep, whereas the Finnish and the remaining Norwegian reindeer isolates constituted two other and separate clades. The tree is drawn to scale, with branch lengths measured in the number of substitutions per site. [file Image_1.TIF]
